# Ziprasidone and its Association with Sudden Cardiac Death - A Case Report

**Published:** 2004

**Authors:** Ajeet Sidana, B S Chavan, Gurvinder Pal Singh, Divay Mangla

**Affiliations:** 1 Senior Lecturer, Department of Psychiatry, Govt. Medical College & Hospital, Sector - 32, Chandigarh, India; 2 Professor & Head, Department of Psychiatry, Govt. Medical College & Hospital, Sector - 32, Chandigarh, India; 3 Senior Resident, Department of Psychiatry, Govt. Medical College & Hospital, Sector - 32, Chandigarh, India; 4 Senior Resident, Department of Psychiatry, Govt. Medical College & Hospital, Sector - 32, Chandigarh, India

**Keywords:** Ziprasidone, Sudden Cardiac Death

## Abstract

Ziprasidone, an atypical antipsychotic, widely in use because of its better side effect profile. Recently some researchers have raised doubts about their association with sudden cardiac death. Here the authors present such a case report with Ziprasidone in a Schizophrenic patient and it is being suggested that psychiatrist must remain vigilant about cardiac harmful effects with newer antipsychotics for the better care of patient.


Indian Journal of Psychiatry, 2004, 46(I)79-80

### Ziprasidone and its Association with Sudden Cardiac Death - A Case Report

Ajeet Sidana[1*], B.S. Chavan[2], Gurvinder Pal Singh[3], Divay Mangla[4]

**ABSTRACT:**

Ziprasidone, an atypical antipsychotic, widely in use because of its better side effect profile. Recently some researchers have raised doubts about their association with sudden cardiac death. Here the authors present such a case report with Ziprasidone in a Schizophrenic patient and it is being suggested that psychiatrist must remain vigilant about cardiac harmful effects with newer antipsychotics for the better care of patient.

Key Words: Ziprasidone, Sudden Cardiac Death

### INTRODUCTION

Ziprasidone is a novel atypical antipsychotic with unique pharmacological profile and was approved by FDA in Feb. 2001 for the treatment of psychotic disorders. It is used widely because of better side effect profile. In recent years, concern about reports of sudden unexpected death occurring in psychiatric patients being treated with atypical antipsychotics has been raised (Haddad et al, 2002). The possibility of a causal link has been a controversial area and need further research to quantify the risk of sudden death with atypical antipsychotics. Here, we are presenting a case report of a psychotic female patient who was taking Ziprasidone for one month and she died suddenly in the psychiatric ward.

### CASE REPORT

Mrs. S. a 44 years old woman suffering from schizophrenia for last 2 years was referred to psychiatry from medical emergency. She was admitted in the Psychiatry ward with complaints of talking irrelevantly, oddities of behaviour, poor self-care, poor socialization and suicidal ideas. Mental examination showed somatic delusions, blunt affect and poverty of content of speech. Patient had no previous history of cardiovascular disorders (Hypertension,arrythmias,other medical illnesses or neurological disorders). Patient was receiving 120 mg/day of Ziprasidone for last one month and had also received three modified ECT after pre-anaesthetic check-up, two months before from a private psychiatrist. There was no ECT related side effects and patient did not show any improvement with ECT . After admission in psychiatry ward, all routine investigations (Haemogram, Serum electrolytes, RFT & TFT) were within normal limits.

The dose of Ziprasidone was increased to 160 mg/day and within 4 days, patient showed improvement in communication, oral intake, self care and became cooperative. On 5th day , she was asked to have a stroll within the ward. After 10-15 steps, she suddenly fell down on the ground in front of the nursing station and turned unconscious with great difficulty in respiration. On examination, patient was unresponsive and had fast, feeble, low volume pulse which otherwise appeared regular and blood pressure was unrecordable. All emergency resuscitative measures were tried by the anesthetist and the physician, but despite all resuscitative interventions patient could not be revived. Apparently, patient did not have underlying physical or neurological disorder leading to sudden death. However, such a conclusion was difficult to be drawn in the absence of autopsy findings. It is possible that the sudden death might have been caused by Ziprasidone leading to ventricular tachyarrhythmias after the dose was increased to 160 mg/day . It may be hypothesized that patient might had been at risk because of some underlying sub-clinical cardiac pathology, which was probably missed and sudden death was the result of an adverse cardiovascular event viz. tachyarrhythmia induced by Ziprasidone. Even though sudden death with Risperidone (Ravin et al,1997) and serious cardiac problems with clozapine (Modai et al,2002) has been reported, this is the first such report of Ziprasidone associated sudden cardiac death. All antipsychotic drugs have the potential for serious adverse events. Balancing these risks with the positive effects of antipsychotic drugs poses a challenge for psychiatry (Glassman et al, 2001). Ziprasidone does prolong

[1]Senior Lecturer
[2]Professor & Head
[3]Senior Resident
[4]Senior Resident
*Department of Psychiatry , Govt. Medical College & Hospital, Sector – 32, Chandigarh e-mail. ajeetsidana@hotmail.com the QTc interval but more evidence is required to establish that it can lead to torsade de pointes or sudden death (Taylor, 2003).

It is being suggested that more research is needed to prove if Ziprasidone is entirely safe and meanwhile the base line ECG and cardiologist opinions may be sought in patients who are above 40 years of age. The dose of Ziprasidone may be titrated more slowly . Also the coprescription of ziprasidone with other drugs that prolong the QT interval should be avoided (T aylor,2003). The mental health professional should be made aware of cardiac risk associated with Ziprasidone, till it is concluded that it is safe.

### Limitation

Base line ECG was not done to comment on the pre-treatment status of cardiac functioning.

### REFERENCES

Glassman ,H.A.,Bigger, T.J.(2001) Antipsychotic drugs: Prolonged QT c interval,Torsade de pointes,and sudden death.Am.J.Psychiatry , 158,1774-1782.

Haddad, M.P ., Anderson, M.I. (2002) Antipsychotic-Related QT c prolongation, Torsade de pointes and sudden death. Drugs, 62(1 1),1649-1671.

Modai ,I.,Hirschmann ,S.,Rava ,A.,Kurs, R.,Barak, P .,Lichtenberg, P.,Ritsner, M.(2002) Sudden death in patients receiving clozapine treatment:a preliminary investigation.J clin Psychopharmacol,20,325-327.

Ravin, D.S.,Levenson, J.W .(1997) Fatal cardiac event following invitation of risperidone therapy . Ann. Pharmacother, 31, 867-870.

Taylor, D.(2003) Ziprasidone in the management of schizophrenia: the QT interval issue in con text. CNS Drugs, 17(6), 423-30.

